# Spatial distribution differences of 25-hydroxyvitamin D in healthy elderly people under the influence of geographical environmental factors

**DOI:** 10.1038/s41598-022-17198-9

**Published:** 2022-07-27

**Authors:** Wenjie Yang, Miao Ge, Yabo Wang, Xinrui Pang, Congxia Wang

**Affiliations:** 1 Institute of Health Geography, School of Geographical Sciences and Tourism, Shaanxi Normal University, Xi’an, 710119 Shaanxi China; 2 The Second Affiliated Hospital of Xi’an Jiaotong University, Xi’an, 710119 Shaanxi China

**Keywords:** Environmental sciences, Environmental social sciences, Endocrinology

## Abstract

The main targets of this were to screen the factors that may influence the distribution of 25-hydroxyvitamin D[25(OH)D] reference value in healthy elderly people in China, and further explored the geographical distribution differences of 25(OH)D reference value in China. In this study, we collected the 25(OH)D of 25,470 healthy elderly from 58 cities in China to analyze the correlation between 25(OH)D and 22 geography secondary indexes through spearman regression analysis. Six indexes with significant correlation were extracted, and a ridge regression model was built, and the country’s urban healthy elderly’25(OH)D reference value was predicted. By using the disjunctive Kriging method, we obtained the geographical distribution of 25(OH)D reference values for healthy elderly people in China. The reference value of 25(OH)D for healthy elderly in China was significantly correlated with the 6 secondary indexes, namely, latitude (°), annual temperature range (°C), annual sunshine hours (h), annual mean temperature (°C), annual mean relative humidity (%), and annual precipitation (mm). The geographical distribution of 25(OH)D values of healthy elderly in China showed a trend of being higher in South China and lower in North China, and higher in coastal areas and lower in inland areas. This study lays a foundation for further research on the mechanism of different influencing factors on the reference value of 25(OH)D index. A ridge regression model composed of significant influencing factors has been established to provide the basis for formulating reference criteria for the treatment factors of the vitamin D deficiency and prognostic factors of the COVID-19 using 25(OH)D reference value in different regions.

## Introduction

Vitamin D is a fundamental regulator of host defenses by activating genes related to innate and adaptive immunity. It not only boosts a person's innate immune system, but also prevents innate immune system from becoming overactive. After analyzing data from the global COVID-19 pandemic, a team of researchers led by Northwestern University found a strong correlation between severe vitamin D deficiency and COVID-19 deaths^1^. The report showed that a high positive correlation between vitamin D levels and cytokine storms, and vitamin D deficiency was also found to be associated with death. Cytokine storms are caused by an overreaction of the immune system, and it can severely damage the lungs, causing acute respiratory distress syndrome and patient death, which appears to be primarily responsible for the deaths of COVID-19 patients.

Vitamin D, also known as the sunshine vitamin, is mainly derived from the self-synthesis of the skin under sunlight, and a small part comes from food intake. This is due to the fact that sunlight is rich in ultraviolet rays. Ultraviolet rays are classified as ultraviolet radiation *a* (UVA),ultraviolet radiation *b* (UVB), and ultraviolet radiation *c* (UVC)depending on the wavelength. UVB greatly promotes the body's synthesis of vitamin D. Therefore, vitamin D is also a good index for solar UVB exposure. Vitamin D and UVA from sunlight also increase blood nitric oxide levels, which has many beneficial effects including reduced risk of infectious diseases, cardiovascular disease, and high blood pressure, in a seasonal manner^2–8^.

The value of serum 25-hydroxy vitamin D[25(OH)D] is the best index to evaluate the level of vitamin D. However, throughout the previous studies, it can be found that there were obvious regional differences in the reference value of serum 25-hydroxy vitamin D^9^. The geographical environment may be the factor affecting the distribution of 25(OH)D reference value^10^. The relationship between health and living environment is mainly evaluated from natural environment and social environment^11^. Through regional comparison and case–control methods, a large number of studies have been carried out on the relationship between health, disease, natural and social environmental factors. Among them, the natural environment has a significant impact on the reference value of various medical indicators^12^.

Therefore, from the point of view of natural environment, this paper constructed an index system to screen the factors that may affect serum 25(OH)D reference value in Chinese healthy elderly. The reference value of serum 25(OH)D of the elderly in different regions of China was predicted by constructing a model. Geostatistical analysis was used to explore the distribution trend of serum 25(OH)D reference value. In the end, the Influence of geographic environmental factors on the distribution of vitamin D reference value in healthy elderly people was explored.

## Methods

### Data collection methods

#### 25(OH)D reference value data source

Useing serum 25(OH)D as the keyword for subject searched in China national knowledge infrastructure (CNKI), Wanfang Scientific Journal Full-text Database, and PubMed Database, respectively. The total of 25,470 cases of serum 25(OH)D values from elderly people over 60 years old were collected (The samples were distributed in 23 provinces, 5 autonomous regions, 4 municipalities, and 1 special administrative region, lack of Macao and Taiwan). Among them, 12,863 were males (50. 5%), and 12,607 were females (49. 5%). People who suffered from cancer, diabetes, osteoporosis, fractures, endocrine-related metabolic disorders or those who took drugs that affect 25(OH)D value were excluded. The selected subjects were all ethnic groups of Han nationality. The unit was ng/ml. This study of patient specimens was approved by the ethical committee of Shaanxi Normal University, in compliance with the guidelines of the 1975 Declaration of Helsinki. All data were experimental data that obtained from published articles, which displayed in the Appendix. In order to protect the legitimate rights and interests of subjects and researchers, and to ensure the science and reliability of the research, informed consent was signed by the subject population or their families.

#### Construction of index system

We selected spatial location, terrain indicators, climate, and soil properties as geographic indicators, and subdivided them into 22 sub-indices (Table 1). The location indicators came from the National Bureau of Surveying and Mapping (http://www.nasg.gov.cn/). The climate indicators were selected from the China Meteorological Science Data Sharing Service Network (http://cdc.cma.gov.cn/). The soil indicators derived from the Harmonized World Soil Database (HWSD) (http://www.fao.org/nr/land/soils/harmonized-world-soil-database/zh/).

**Table 1:** The geographic indicators.

Type	The name and unit of the indicator	Type	The name and unit of the indicator
Location	Longitude (°)	Soil	Reference bulk density of topsoil (kg/dm^3^)
	Latitude (°)		Gravel content of topsoil (% vol)
Terrain indicators	Altitude (m)		Organic matter content of topsoil (% wt)
Climate	Annual sunshine duration (h)		pH value of topsoil
	Annual mean temperature (°C)		Cation exchange capacity of topsoil (cmol/kg)
	Annual mean relative humidity (%)		Base saturation of topsoil (%)
	Annual recipitation (mm)		Total exchangeable capacity of topsoil (cmol/kg)
	Annual temperature range (°C)		Calcium carbonate content of topsoil (%)
Soil	Percentage of sand in topsoil (% wt)		Calcium sulfate content of topsoil (%)
	Topsoil silt percentage (% wt)		The alkalinity of topsoil (cmol/kg)
	Percentage of clay in topsoil (% wt)		The salinity of topsoil (dS/m)

### Data analysis methods

#### Spatial autocorrelation analysis

The spatial autocorrelation of the sample data were analyzed by ArcGIS 10.2 software. The correlation between the value and the spatial position was determined by outputting the value of Mordan’s *I*, *Z* score^13,14^. The formula for calculating the Moran's *I* is as follows (1).

1 $$ I = \frac{{n\sum\nolimits_{i = 1}^{n} {\sum\nolimits_{j = 1}^{n} {w_{ij} } \left( {y_{i} - \overline{y}} \right)\left( {y_{j} - \overline{y}} \right)} }}{{\left( {\sum\nolimits_{i = 1}^{n} {\sum\nolimits_{j = 1}^{n} {w_{ij} } } } \right)\sum\nolimits_{i = 1}^{n} {\left( {y_{i} - \overline{y}} \right)^{2} } }} $$

where *y*_*i*_ represents the attribute value of the spatial variable in the *i* region, *y*_*j*_is the spatial variable attribute value in the* j* region, *n* and *w*_*ij*_ represent the number of sample points and the spatial weight matrix element, respectively.

*Z* score formula is as follows (2).

2 $$ Z = \frac{I - E\left( I \right)}{{\sqrt {Var\left( I \right)} }} $$

#### Correlation analysis

Correlation analysis was applied to determine whether there were correlations between geographical environment factors and serum 25(OH)D reference value^15^. SPSS 22.0 software was used to analyze the correlations between the reference value and 22 geographical factors. The correlation coefficient of Spearman grade was selected, and the expression of the Spearman grade correlation coefficient is as follows (3).

3 $$ r = 1 - \frac{{6\sum\nolimits_{i = 1}^{n} {d_{i}^{2} } }}{{n\left( {n^{2} - 1} \right)}} $$

#### Models

##### Establish predicted models

*Ridge regression analysis* Ridge regression analysis is an improved least square method, which is more in line with the actual situation^16^. The SAS 12.0 software was employed to establish the model. The relevant geographical factors were taken as independent variables, and the reference value of 25(OH)D was used as dependent variable. The geographical factor data of 2322 cities and counties in China were inputted into the model, and finally the predicted value of serum 25(OH)D of 2322 cities and counties in China was obtained.

*Support vector machines* Support vector machine (SVM) is a machine learning method with a high proportion of applications, which is widely used in many fields^17^. This method uses the appropriate kernel function to transform the problem reasonably and can solve the problem of linear classification. Different kernel functions are used to obtain prediction data, which can be mapped to high-dimensional space. This method requires four different kernel functions to implement by using Clementine 12.0 software.

The formula of linear kernel function is as follows.

4 $$ K\left( {x,y} \right) = x \cdot y $$

The formula of polynomial kernel function is as follows.

5 $$ K\left( {x,y} \right) = \left[ {\left( {x \cdot y} \right) + 1} \right]^{d} $$

The formula of RBF kernel function is as follows.

6 $$ K\left( {x,y} \right) = \exp \left[ { - {{\left| {x - y} \right|^{2} } \mathord{\left/ {\vphantom {{\left| {x - y} \right|^{2} } {\sigma^{2} }}} \right. \kern-\nulldelimiterspace} {\sigma^{2} }}} \right] $$

The formula of Sigmoid kernel function is as follows.

7 $$ K\left( {x,y} \right) = \tanh \left[ {v\left( {x \cdot y} \right) + b} \right] $$

##### Models select and test

Taylor diagram^18^ is often used to evaluate the accuracy of models^19^. The scatter in the Taylor diagram represents the model, the solid lineis the correlation coefficient, the horizontal and vertical axis represents the standard deviation, and the dotted line is the root mean square error. Wilcoxon Rank Sum test is often used to judge whether there is the significant difference between the predicted data and the measured data. It does not require pairwise data to follow normal distribution^20^. When *P* > 0. 05, it is considered that there is no significant difference, which indicates that the predicted value is in good agreement with the measured value.

##### Model prediction and geostatistical analysis

The spatial trend analysis and the Kriging mapping of the predicted data were carried out by using ArcGIS 10.2. The predicted value in different locations were modeled by variation function and Kriging so as to realize the continuous distribution of predicted values^21^. By using the model interpolation, the geographical distribution map of serum 25(OH)D reference value of healthy Chinese elderly can be constructed, which will be helpful to further analyze the regional differences in space.

### Statement

All methods were carried out in accordance with relevant guidelines and regulations. Informed consent was obtained from all subjects and legal guardian(s).

### Approval for human experiments

This study of patient specimens was approved by the ethical committee of Shaanxi Normal University, in compliance with the guidelines of the 1975 Declaration of Helsinki. All data were experimental data obtained from published articles. Literature for data sources is in the Appendix. Informed consent was signed by the patients and their families.

## Results

### Spatial autocorrelation analysis

The Moran index (Moran *I*) was 0.823 (> 0), and the global autocorrelation index *Z* was 4. 095 (> 2. 58). The probability value *P* was 0. 000, which indicated that there was significant spatial heterogeneity in serum 25(OH)D reference value.

### Correlation analysis

Through Spearman correlation analysis, the 25(OH)D reference value of healthy elderly in various regions of China and the geographical factors were obtained. The value of correlation coefficient (r) and significance coefficient (*P*) were used to judge the correlation between geographic factors and the 25(OH)D reference values. If *P* ≤ 0.01, they are significantly correlated. If 0.01 < *P* ≤ 0.05, they are correlated, and P ≥ 0.05, the correlation between the them is not significant. Through the values, it can be clearly found that there were 6 geographical factors that have a correlation with serum 25(OH)D value (Table 2).

**Table 2:** Results of correlation analysis.

Symbol	Geographic factors	*R* value	*P* value
X_1_	Latitude (°)	− 0. 27**	0. 005
X_2_	Annual sunshine duration (h)	0. 36**	0. 000
X_3_	Annual mean temperature (°C)	0. 21*	0. 029
X_4_	Annual mean relative humidity (%)	0. 26**	0. 008
X_5_	Annual precipitation (mm)	0. 24*	0. 01
X_6_	Annual temperature range (°C)	− 0. 20*	0. 04

*Represents correlation, **represents the significant correlation.

### Models

#### Ridge regression analysis

**Figure 1:**
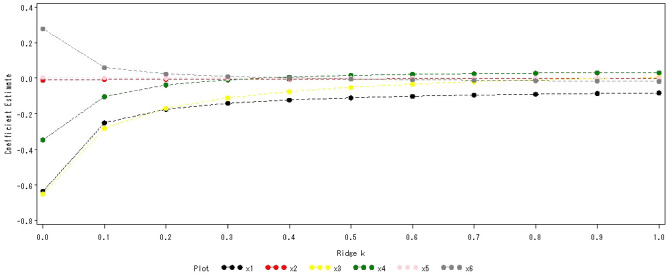
Ridge trace map of serum 25(OH)D reference value.

The above 6 geographical factors were used as independent variables, and the reference value of serum 25(OH)D were used as dependent variables. The horizontal axis represented the ridge trace parameters, and the vertical axis represented the regression coefficient of each factor (Fig. 1). When the ridge parameter *K* = 0. 3, the trend of the ridge trace was relatively stable, and the regression equation was obtained as follows.

$$ {\hat{\text{Y}}} = {56}.{ 51} - 0.{\text{25X}}_{{1}} - 0.00{\text{75X}}_{{2}} - 0.{\text{28X}}_{{3}} - 0.{1}0{\text{X}}_{{4}} + 0.00{\text{19X}}_{{5}} + 0.0{\text{59X}}_{{6}} \pm {9}.0{6}. $$

In this regression equation, ${\hat{\text{Y}}}$ represented the 25(OH)D value (ng/ml), and 9. 06 was the residual standard deviation. The geographic data of 2322 cities and counties across the whole country were inputted into the ridge regression equation. The predicted value of 25(OH)D value in the serum 25(OH)D value of the elderly in 2322 of the cities and counties across the country were exported.

#### Support vector machines

**Figure 2:**
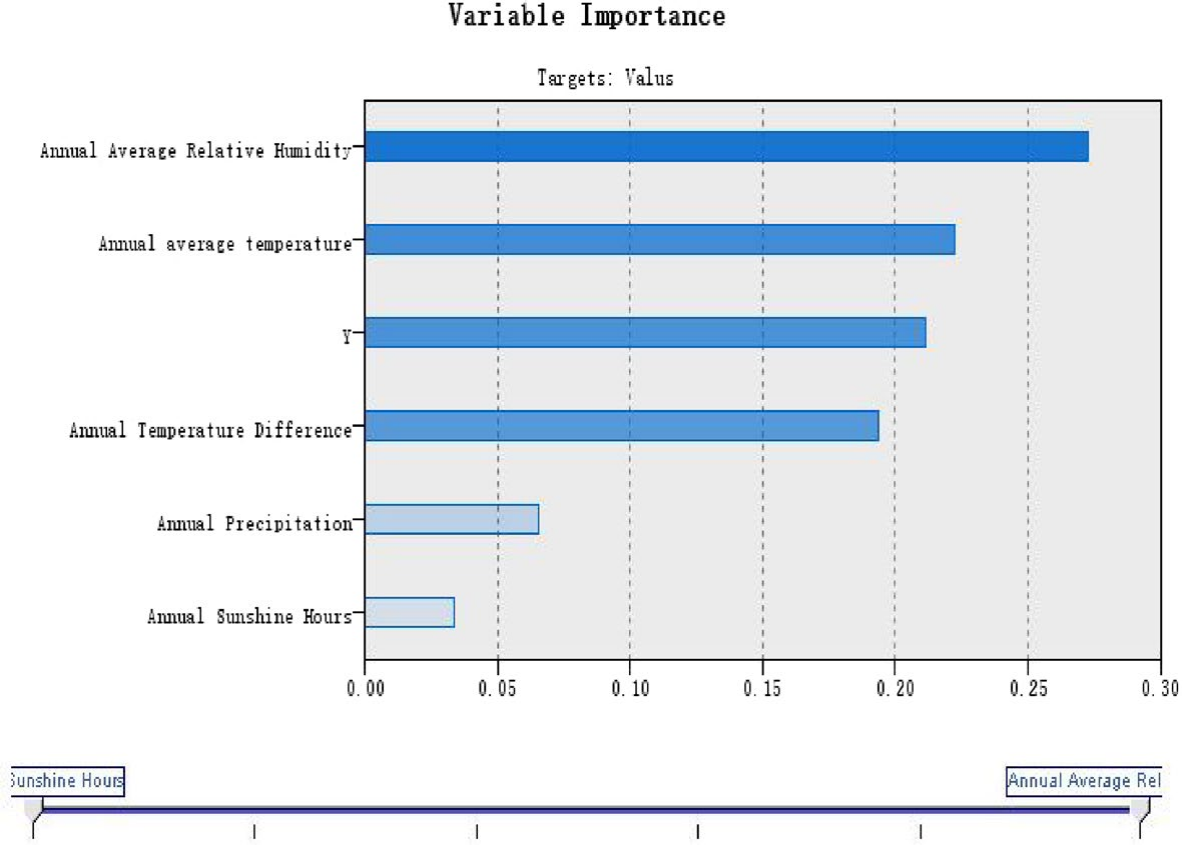
Linear model of serum 25(OH)D reference value in healthy elderly.

**Figure 3:**
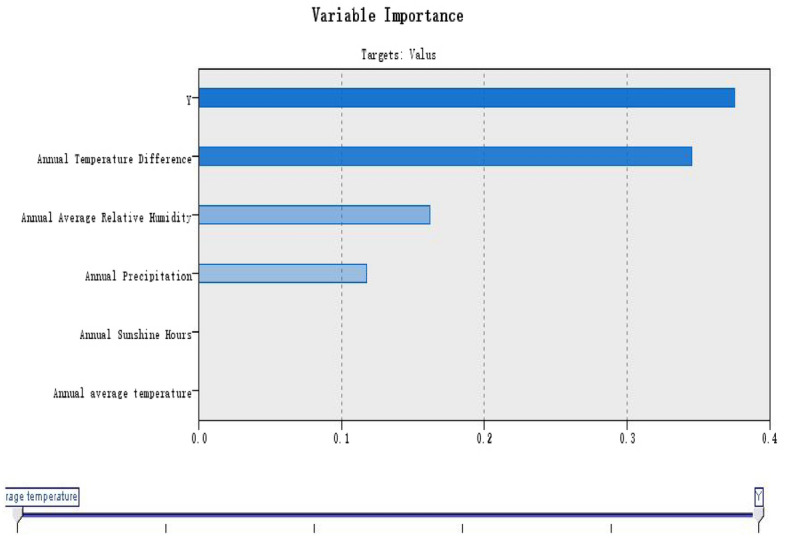
Ploynomial model of serum 25(OH)D reference value in healthy elderly.

**Figure 4:**
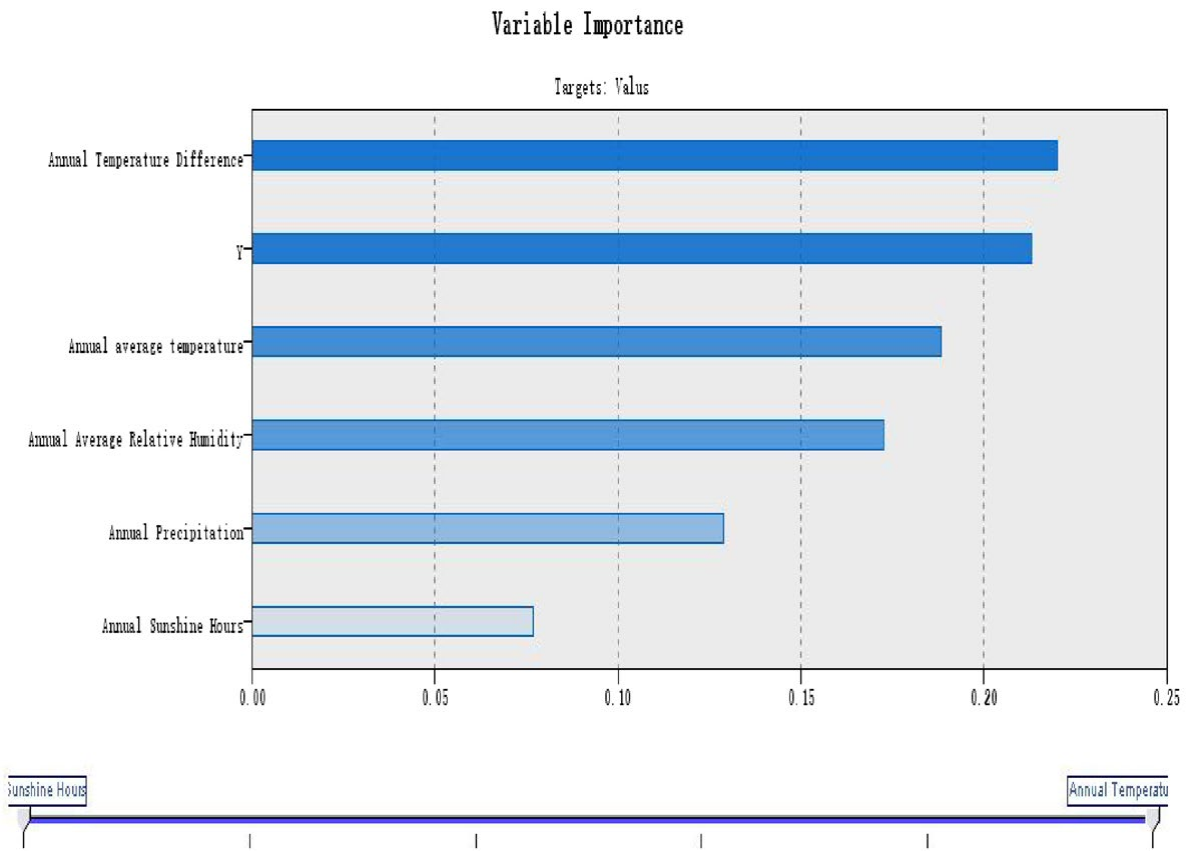
RBF model of serum 25(OH)D reference value in healthy elderly.

**Figure 5:**
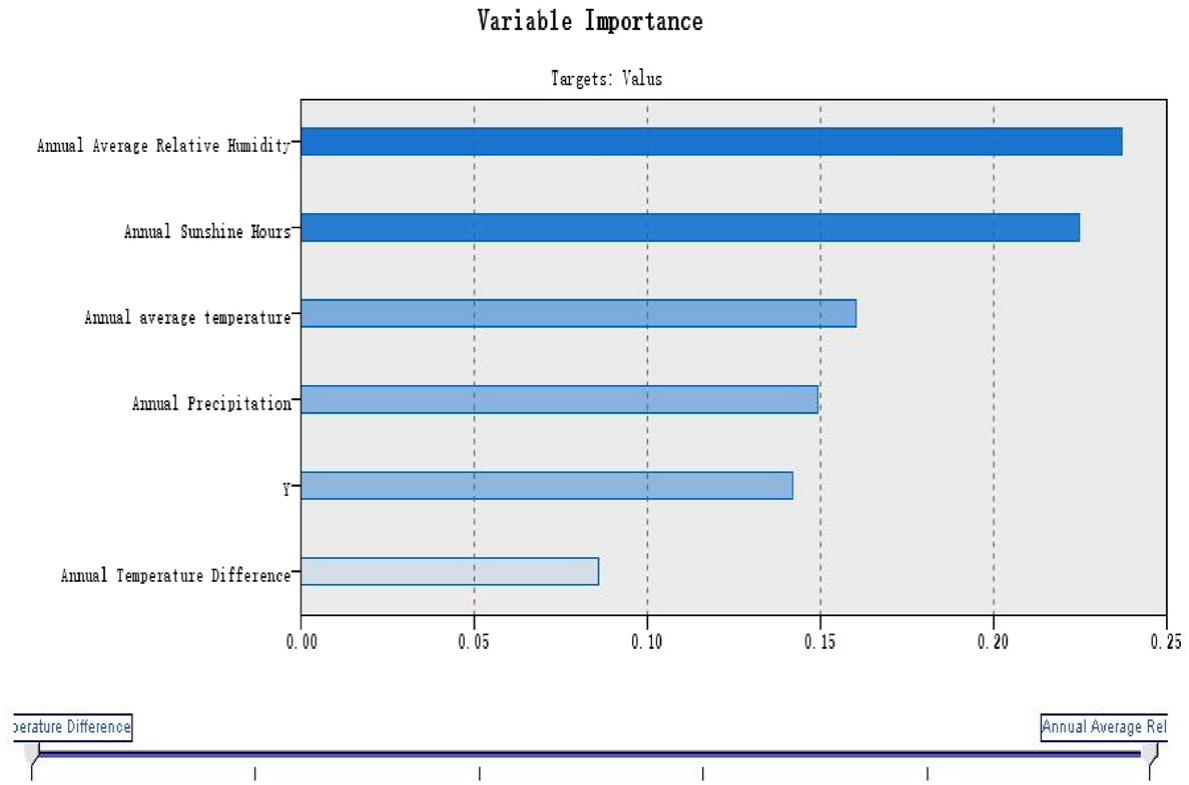
Sigmoid model of serum 25(OH)D reference value in healthy elderly.

The relevant geographical factors were used as input variables and serum 25(OH)D reference value as output variables for machine learning. Four kinds of expert kernel functions including RBF kernel function, polynomial kernel function, Sigmoid kernel function and linear kernel function were trained in turn, and four models were obtained respectively (Figs. 2, 3, 4, 5).

#### Models select and test

##### Models select

**Figure 6:**
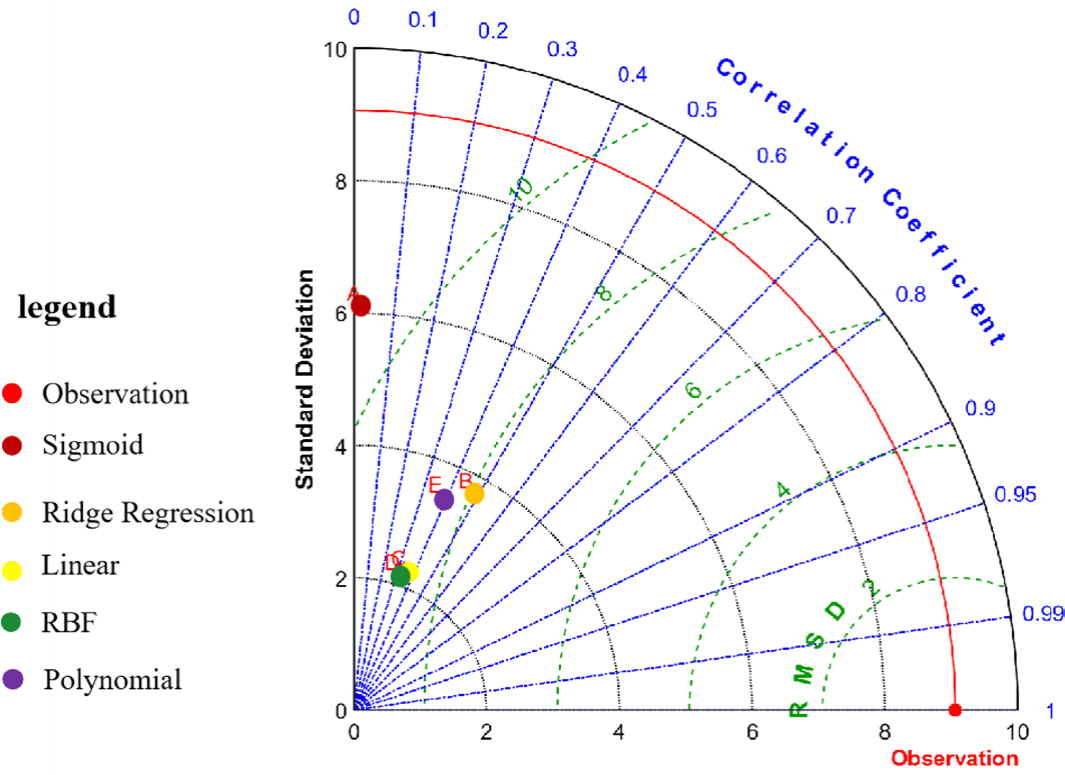
Taylor Diagram representation of the accuracy of different models.

The evaluation principle of the best model was that the greater the correlation coefficient between the predicted value of the model and the measured value, the smaller the ratio of the root mean square error to the measured standard deviation, and the closer the ratio of the standard deviation to the measured standard deviation^22^. The parameters of the Taylor diagram were showed in Table 3. The Taylor diagram of the 25(OH)D reference value predicted by the five models was shown in Fig. 6. The resuls showed that model B (Ridge Regression) was the best fit.

**Table 3:** Prediction model error to each kernel function of serum 25(OH)D reference value.

Symbol	Model name	RMSE (E)	Standard deviation (SD)	Correlation coefficient (CC)
A	Sigmoid	10. 8	6. 10	0. 02
B	Ridge regression	9. 06	3. 74	0. 30
C	Linear	8. 99	2. 25	0. 13
D	RBF	8. 98	2. 15	0. 11
E	Polynomial	8. 37	3. 48	0. 22

##### Model test

The Ridge Regression model was selected to predict serum 25(OH)D reference value. The results showed that *P* = 0.79(> 0. 05), indicating that there was no significant difference between the predicted values and the measured values.

#### Spatial distribution of reference value

##### Geostatistical analysis

**Figure 7:**
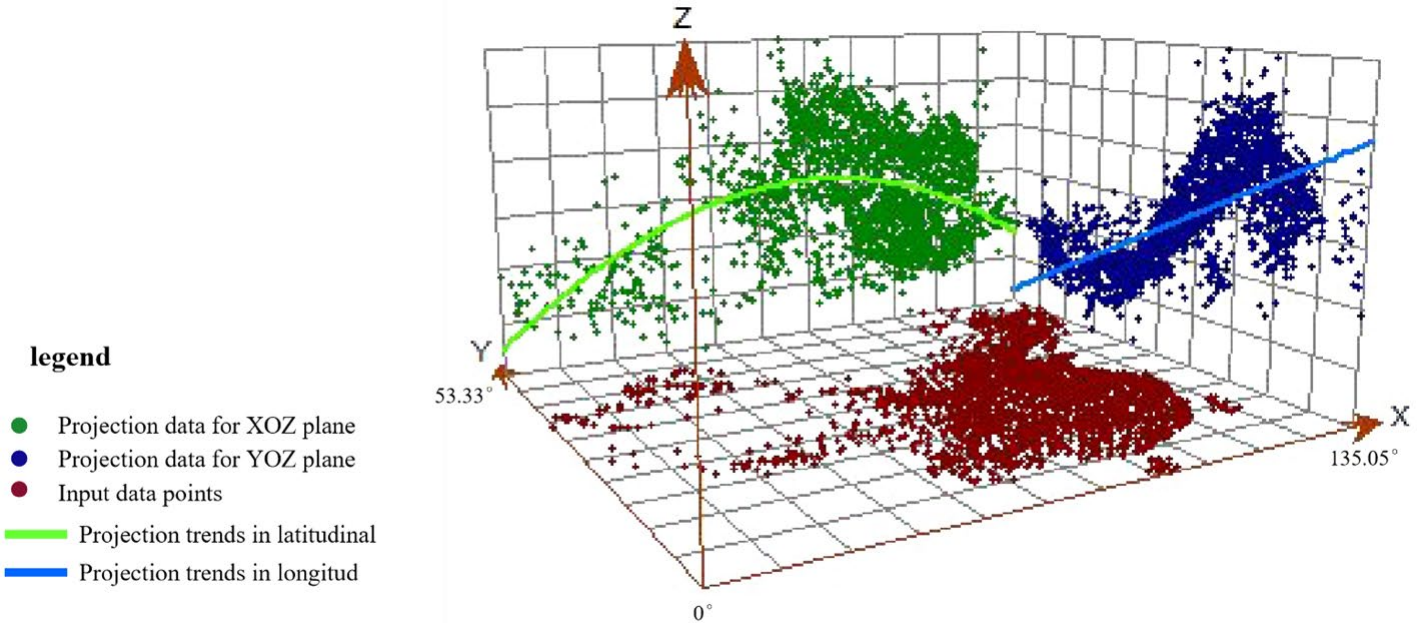
Spatial distribution trend of serum 25(OH)D value in Chinese healthy elderly.

**Figure 8:**
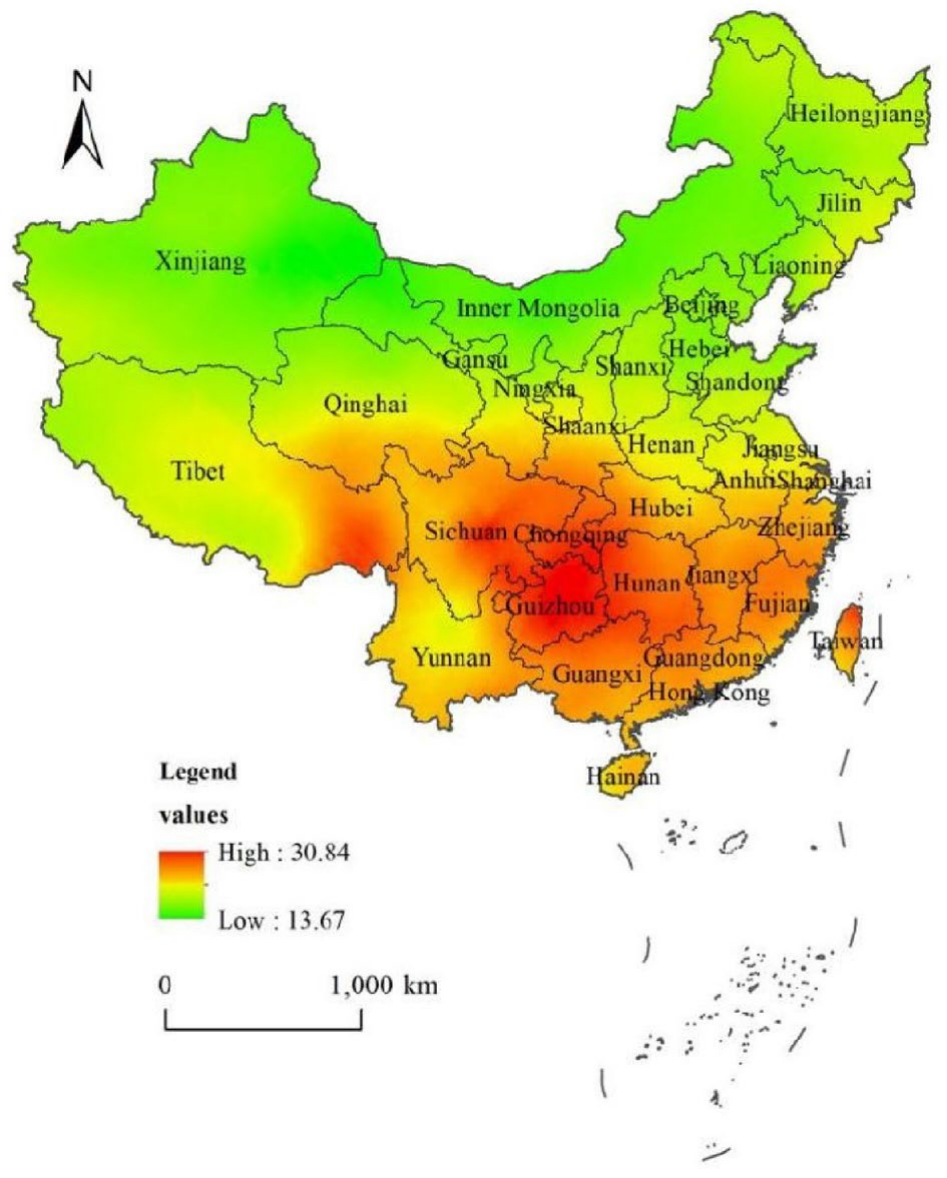
Spatial distribution of serum 25(OH)D reference value in Chinese healthy elderly.

Trend surface analysis was applied to reveal the trend of distribution difference in serum 25(OH)D reference value. From the east to west, the reference value of serum 25(OH)D increased at first and then decreased. And it decreased gradually from south to north (Fig. 7). The change range in the north–south direction (Y axis) was slightly larger than that in the east–west (X axis) direction, which showed a second-order change. The data were tested by K-S test, and the results indicated that the data didn't have the characteristics of normal distribution (*P* < 0. 01). The Kriging spatial interpolation method was used to make the spatial distribution map of serum 25(OH)D reference value (Fig. 8). It showed that there was a significant difference in the spatial distribution of serum 25(OH)D in Chinese healthy elderly. The geographical distribution of 25(OH)D value of healthy elderly in China showed a distribution difference trend of being higher in South China and lower in North China, and higher in coastal areas and lower in inland areas.

## Discussion

A large number of epidemiological studies have shown that vitamin D deficiency has become a global health problem. It is estimated that 22% of the people in the United States, Canada and Europe lack vitamin D or vitamin D deficiency^23–27^. In China, previous reports of 25(OH)D_2_ and 25(OH)D_3_ levels in 23,695 patients requiring a 25(OH)D test showed a vitamin D deficiency rate of 84.1%^28^. Therefore, in clinical practice, the early detection and management of vitamin D deficiency has become a growing concern.

In this study, we found that the geographical distribution of 25(OH)D value of healthy elderly in China showed a distribution difference trend of being higher in South China and lower in North China, and higher in coastal areas and lower in inland areas. A study from the vitamin D status of healthy people in Sichuan Province showed that 35.5% and 38.6% of the total samples were found to have vitamin D deficiency and deficiency, but 25.9% of the participants had adequate vitamin D. The adequacy rate of the Sichuan study was higher than a previous report of 23,695 patients from Beijing, China, showing only 15. 5% adequacy^29^. This is consistent with our results, in which the reference value of serum 25(OH)D in the south is higher than that in the north. This difference may be caused by a variety of factors.

Vitamin D, also known as "sunshine vitamin", is mainly derived from the self-synthesis of the skin under ultraviolet radiation *b*(UVB), and a small part comes from food intake^30^. Sunshine exposure and vitamin D intake are the main determinants, but these are modified by other factors. It is worth noting here that UVB wavelengths of solar radiation can serve as an etiological factor in melanoma genesis, it must be acknowledged that it is also necessary for vitamin D formation that can not only act as a protector against UVR, but also has a role in attenuating carcinogenesis and tumor progression^31–36^.

In China, the sunlight, climate and soil conditions are different in different regions. All these factors are likely to affect or be affected by vitamin D status^37^. We, therefore, investigated whether variability of geographical environment factors in China necessarily confer adequate vitamin D optimization among apparent healthy elderly in different geographical sections. We mainly explored the factors associated with serum 25(OH)D, and we found the reference value of 25(OH)D of healthy elderly in China was significantly correlated with the 6 secondary indexes, namely, latitude (°), annual temperature range (°C), annual sunshine hours (h), annual mean temperature (°C), annual mean relative humidity (%) and annual precipitation (mm).

Areas with more annual sunshine duration (h) have more sun exposure. The higher the latitude varies, the larger the angle of solar altitude varies. The higher the latitude, the longer it takes for UVB to pass through the atmosphere and the less the amount of UVB reaching the surface. Many studies supported the effect of latitude on the reference value of serum 25(OH)D^38–40^. Low 25(OH)D concentrations were found to be more common in high-risk populations, such as the elderly, and people with colored skin living in high-latitude countries^41^.

These factors, such as annual mean temperature (°C), annual mean relative humidity (%), and annual precipitation (mm), will affect people's dressing habits. The dressing habits of people in areas with large annual temperature are different from those in areas with small. When the temperature is low, people wear thicker clothes, and the skin will be less likely to be exposed to UVB, which affects the synthesis of serum vitamin D. In areas with higher annual mean temperatures, people wear thinner clothes all year round, and the skin area will be more likely to be exposed to UVB, which is conducive to the synthesis of serum vitamin D^42^.There are low latitudes in the southwest and southeast regions, and the temperature difference between them is relatively small. The skin of humans has more opportunities to be exposed to UVB. The northeast and northwest regions have high latitudes and large temperature differences. The skin of humans has fewer opportunities be exposed to UVB than others. All the above factors have an indirect effect on the synthesis of vitamin D, which leads to this spatial distribution difference in serum 25(OH)D of healthy elderly people in China.

Vitamin D can also be obtained from foods such as fatty fish(e.g., salmon and tuna)^43^. These foods are abundant in southern coastal cities and less abundant in inland cities. Differences in eating habits between the north and the south people may also account for the distribution.

The reference value of vitamin D belongs to the category of medical research.But in this research,we used geographical analysis to study it. The medical reference values were expressed by Kriging interpolation and expressed in different colors on the map according to the values. It will make it more convenient for us to analyze the differences in reference values of vitamin D on the map.There were many studies on the effects of geographical factors such as latitude and light on vitamin D, mainly focusing on the correlation between them. But in our research, we not only study the correlation but also use the related geographical factors to construct the model. By using this model, we can conveniently calculate the reference value of 25(OH)D of a place when we know the geographical environment factors of a place.More importantly, we introduce the Taylor diagram method to measure the accuracy of the ecological model into the comparison of the accuracy of the medical reference model and optimize the method of model screening.

There are still some shortcomings in this study. First, in the selection of population characteristics and environmental factors, we did not consider the influence of physical activities and some special pollutants on human serum 25(OH)D, which would introduce irreversible errors to the results. Second, we only used a national cross-sectional study and environmental data corresponding to the testing time. The study did not consider the environmental lag of one season or more, so it could not determine the short-term effect in terms of time, which may bring errors. Future studies will need add cohort data to study the time lag, and determine dietary habits and exercise status through questionnaires to control confounding factors more comprehensively.

## Conclusions

The reference value of 25(OH)D in the Chinese elderly is related to 6 geographical factors. The ridge regression model established in this study can predict the reference value of 25(OH)D in different regions. If the latitude (°), annual temperature range (°C), annual sunshine duration (h), annual mean temperature (°C), annual mean relative humidity (%), and annual precipitation (mm) are known in a certain area. According to the equation:

$$ \hat{Y} = 56.51 - 0.25X_{1} - 0.0075X_{2} - 0.28X_{3} - 0.10X_{4} + 0.0019X_{5} + 0.059X_{6} \pm 9.06. $$

The 25(OH)D reference value can be predicted.

Vitamin D in China has a spatial distribution differences trend of high in the south and low in the north. The elderly in the North should pay more attention to vitamin D supplements.

## Supplementary Information

Supplementary Information.

## Data Availability

The data that support the findings of this study are openly available in China national knowledge infrastructure (CNKI), Wanfang Scientific Journal Full-text Database, and Pub Med Database. They are available from the published literature from these Database. The titles of these literature are in the Appendix.
