# Supplementary material for: Spatial distribution differences of 25-hydroxyvitamin D in healthy elderly people under the influence of geographical environmental factors
Source: Sci Rep. 2022 Jul 27;12:12781. doi: 10.1038/s41598-022-17198-9 (PMC9326152; doi:10.1038/s41598-022-17198-9)
Supplement: Supplementary file 1 — Supplementary Information. [file 41598_2022_17198_MOESM1_ESM.docx]

**Appendix of data sources**

1. Jie, C.et al. Changes and clinical significance of serum 25-hydroxyvitamin D and peripheral blood Nlr in patients with acute coronary syndrome. *Jilin Medicine*. **43**, 378-381 (2022).
2. Gong, C.et al. Relationship between 25(OH)D level and ischemic white matter lesions in the elderly. *Medical Information*. **35**, 137-139 (2022).
3. Yang, G.et al. Relationship between serum vascular endothelial cadherin and 25- hydroxyvitamin D levels and the condition and prognosis of patients with acute ischemic stroke. *Journal of Integrated Chinese and Western Medicine on Cardiovascular and Cerebrovascular Diseases*. **20**, 931-936 (2022).
4. Zhang, C.et al. Study on the relationship between fasting C-peptide and 25- hydroxyvitamin D levels and bone mineral density in elderly patients with type 2 diabetes. *Journal of PLA Medicine*. **34**, 83-86 (2022).
5. Qiao, Z.et al. Dose-response meta-analysis of serum 25(OH)D level and risk of dementia. *Chinese Journal of Difficult and Complicated Cases.* **21**, 297-301 (2022).
6. Ao W.et al.A comparative study of calcium combined with calcitriol in the treatment of postmenopausal osteoporosis. *Journal of Clinical Psychosomatic Diseases*. **27**, 27-30 (2021).
7. Bian P.et al. Effect of vitamin D_2 injection on serum parathyroid hormone in elderly male patients with vitamin D deficiency. *Chinese Journal of New Drugs and Clinical Medicine*. **40**, 520-523 (2021).
8. Jiaoyue, C. et al. Serum 25-hydroxyvitamin D level in early stage of type 2 diabetic nephropathy and its relationship with advanced glycation end products. *Hebei Medical*. **27**, 92-97 (2021).
9. Xin,C.et al. Study on the correlation between plasma 25- hydroxyvitamin D concentration and the risk of sarcopenia in the elderly [Master]: *Jilin University,* 2021.
10. Danger, D.et al. Clinical study on changes of bone metabolism in patients with hyperthyroidism. *Chinese Medicine and Clinical*. **21**, 3283-3285 (2021).
11. Jiaojiao, H. Study on the correlation between vitamin D, Lp-PLA2 and coronary artery disease in patients with type 2 diabetes [Master]: *Dalian Medical University*, 2021.
12. Libing,H.et al.Expression of serum 25-hydroxyvitamin D3 in patients with systemic lupus erythematosus and its relationship with autoantibodies. *Journal of Guangdong Medical University*. **39**, 93-96 (2021).
13. Zuxiu, H .et al. Study on the relationship between serum cystatin C, homocysteine and 25- hydroxyvitamin D and leukoaraiosis. *Electrocardiogram and Circulation.* **40**, 585-589 (2021).
14. Bo, L.et al. Correlation between serum 25- hydroxyvitamin D, cystatin C and the severity of leukoaraiosis. *Journal of Stroke and Neuropathy*. **38**, 311-314 (2021).
15. Zhifang, L.et al. Correlation analysis of serum bone metabolism indexes and osteoporosis in postmenopausal women. *Laboratory Medicine and Clinical*. **18**, 1692-1694 (2021).
16. Lijun, L.et al. Vitamin D level in elderly patients with community-acquired pneumonia and its effect on immune response. *Zhejiang Practical Medicine*. **26**, 99-102 (2021).
17. Tunan,L.et al. Detection of plasma Hcy, 25-(OH)-D and folic acid levels in elderly patients with Parkinson's disease and their relationship with cognitive function. *Journal of Nanchang University (Medical Edition)* .**61**,36-39 (2021).
18. Xinwei,L.et al. Expression of serum 25-hydroxyvitamin D, matrix metalloproteinase inhibitor -1 and granulocyte-macrophage colony-stimulating factor in patients with pulmonary tuberculosis and their relationship with the disease condition. *Chinese Journal of Coal Industry Medicine*. **24**, 137-143 (2021).
19. Ning,W. Correlation between serum parathyroid hormone level of 25- hydroxyvitamin D and the severity of osteoporosis in elderly patients. *Journal of Practical Medical Technology*. **28**, 240-242 (2021).
20. Xiaorong, X.et al. Therapeutic observation of multiple dialysis methods combined with uremia and their effects on Pth and 25- hydroxyvitamin D levels. *Journal of Clinical and Experimental Medicine*. **20**, 1517-1521 (2021).
21. Ruixiang, Y.et al. Relationship between vitamin D level and bone metabolism and blood uric acid in patients with osteoporosis complicated with hyperuricemia. *Chinese Journal of Osteoporosis*. **27**, 244-247 (2021).
22. Huafeng,Y. Relationship between vitamin D receptor gene polymorphism and osteoporosis in Han and She nationalities in Putian [Master]: *Fujian Medical University*, 2021.
23. Zhenya, Z.et al. Value of bone metabolism markers and calcitonin in elderly patients with osteoporosis. *China Health Engineering*. **20**, 473-475 (2021).
24. Fangwei,Z. et al. Predictive diagnostic value of 25-hydroxyvitamin D3 in eosinophilic chronic sinusitis with nasal polyps. *Chinese Journal of Otolaryngology Head and Neck Surgery*. **56**, 1051-1058 (2021).
25. Xuelei ,A.et al. Changes of serum 25- hydroxyvitamin D level in patients with post-stroke fatigue and the effect of vitamin D3 intervention on neurological rehabilitation. *China Journal of Modern Medicine*. **30**, 32-38 (2020).
26. Jianfeng, C.et al. Correlation between serum β-CTX, OC, PINP, 25(OH)D_3 expression and blood glucose and insulin levels in elderly patients with type 2 diabetes. *Chinese Clinical Research*. **33**, 959-962 (2020).
27. Yanjie, C.et al.Expression and clinical significance of serum 25- hydroxyvitamin D in patients with multiple myeloma. *Chinese Medicine*. **15**, 733-736 (2020).
28. Dongju, H.et al. Effect of zoledronic acid on bone mineral density and 25-(OH)D, β-CTX, N-MID in postmenopausal women with osteoporosis. *Electronic Journal of Modern Medicine and Health Research*. **4**, 73-75 (2020).
29. Fangjie, H.et al. Correlation analysis between serum 25- hydroxyvitamin D concentration and Meige syndrome. *Clinical Medicine of China*. **36**, 358-361 (2020).
30. Qiang, H.et al. Influencing factors and clinical prevention of serum Vegf and 25-(Oh)d levels in patients with rheumatoid arthritis. *Chinese Journal of Preventive Medicine*. **21**, 518-522 (2020).
31. Li ,L.et al. Nutritional status of vitamin D in newly diagnosed patients with ankylosing spondylitis and its clinical significance. *Shanxi Medical Journal*. **49**, 378-381 (2020).
32. Xiaofei, L.et al. Clinical value of 25-Oh-D, Anca and Sf in serum of Sle patients. *International Journal of Laboratory Medicine*. **41**, 95-97 (2020).
33. Jingli, L.et al. Relationship between serum amyloid A, cystatin C and 25- hydroxyvitamin D levels and renal function in patients with diabetic nephropathy. *Jiangsu Medicine*. **46**, 604-607 (2020).
34. Dan, S.et al. Correlation between serum 25- hydroxyvitamin D and hypertension and its risk stratification in the elderly. *Chinese Journal of Geriatric Cardiovascular Diseases*. **22**, 983-985 (2020).
35. Wei W.et al. Relationship between serum 25-(Oh)d level and Vdr gene polymorphism and bone erosion in rheumatoid arthritis. *Chinese Journal of Osteoporosis*. **26**, 992-995 (2020).
36. Qiuxia, W.et al. The relationship between N-MID, 25-(OH)VitD, serum Ca~(2+) and bone mineral density and fracture in menopausal patients with hip fracture caused by OP. *Laboratory Medicine*. **38**, 478-479 (2020).
37. Xue ,Y. Discussion on the relationship between the change of 25- hydroxyvitamin D level and the condition of multiple myeloma. *Chinese Journal of Practical Rural Doctors*. **27**, 59-61 (2020).
38. Min, Z. Changes of 25-hydroxyvitamin D3 and bone metabolism indexes in elderly patients with osteoporosis and their correlation with bone mineral density. *Journal of Naval Medicine.* **41**, 721-723 (2020).
39. Qian ,Z.et al. Changes of serum 1,25- dihydroxyvitamin D3 level in patients with benign primary hyperparathyroidism. *Chinese Journal of Practical Diagnosis and Treatment*. **34**, 66-68 (2020).
40. Yanxu, Z. Measurement and analysis of risk factors related to stress fracture of lower limbs [Master]: *Yan 'an University*, 2020.
41. Hongcai, Z. et al. Clinical significance of combined detection of serum 25-hydroxyvitamin D and tumor markers in diagnosis of colorectal cancer. *Guizhou Medicine.* **44**, 1690-1692 (2020).
42. Bihui, B. et al. Research progress of chronic liver disease and osteoporosis. journal of traditional chinese medicine, *Hunan*. **35**, 144-146 (2019).
43. Xiaoyuan, C.et al. Clinical analysis of serum 25- hydroxyvitamin D level in patients with bullous pemphigoid. *Practical Medicine and Clinical*. **22,** 1262-1265 (2019).
44. Liang, C.et al.Effect of vitamin D on antiplatelet drug reactivity in elderly patients with ischemic stroke. *Clinical Medicine of China*. 300-305 (2019).
45. Shang, D.et al. Effect of vitamin D on 25- hydroxyvitamin D expression and dynamic electrocardiogram in elderly patients with fracture. *Journal of North Sichuan Medical College*. **34,** 59-61 (2019).
46. Jinlong, H.et al. Effect of Dioscorea paste on serum 1,25- dihydroxyvitamin D3 in elderly patients with hypertension. *Clinical Research of Traditional Chinese Medicine*. **11**, 23-26 (2019).
47. Lingling, L.et al.Analysis of the relationship between serum 25- hydroxyvitamin D and renal damage in primary hyperparathyroidism. *Chinese Journal of Integrated Traditional and Western Nephrology*. **20**, 712-714 (2019).
48. Lu, L.et al. Analysis of vitamin nutrition status of inpatients with different types of diseases. *Journal of PLA Medical College*. **40**, 338-341 (2019).
49. Wencai, L. et al. Risk of vitamin D deficiency on chronic diseases of the elderly. *The World's Latest Medical Information Digest.* **19,** 96-97 (2019).
50. Ning, M. et al. Study on the correlation between serum 25- hydroxyvitamin D level and cerebral microvascular disease in the elderly. *Journal of Clinical Military Medical*. **47,** 345-347 (2019).
51. Xiaofei,S.et al. The role of 1,25 dihydroxyvitamin D_3 in the treatment of senile rheumatoid arthritis. *Journal of Henan University of Science and Technology (Medical Edition)*. **37**, 212-215 (2019).
52. Lan, S.et al. Detection and clinical significance of uric acid, 25- hydroxyvitamin D and tumor necrosis factor in patients with nonalcoholic fatty liver disease. *Chinese Journal of Health Laboratory Technology*. **29,** 2491-2493 (2019).
53. Zhihong, Y.et al.Correlation analysis of serum 25(OH)D3 and high-sensitivity C-reactive protein levels with senile type 2 diabetes mellitus complicated with osteoporosis. *Labeled Immunoassays and Clinical Medicine*. **26**, 502-505 (2019).
54. Juan,Y. Research progress of vitamin D deficiency and atrial fibrillation after Cabg [Master]: *Chongqing Medical University*, 2019.
55. Lihua, Z.et al. Correlation between serum 25- hydroxyvitamin D3 and chronic Hbv infection. *Systemic Medicine*. **4**, 45-47 (2019).
56. Tian, Z.et al. Study on the incidence and influencing factors of sarcopenia in elderly patients with type 2 diabetes mellitus. *Practical Geriatrics*. **33**, 772-775 (2019).
57. Xuehua ,Z. Correlation between Cyp24a1 gene polymorphism and colorectal polyps and colorectal cancer [Master]: *Guangxi Medical University*, 2019.
58. Yuqiang, Z.et al. Significance of urinary albumin level in patients with diabetic kidney disease on abnormal bone metabolism. *Chinese Journal of Health Care and Medicine*. **21**, 433-435 (2019).
59. Changda, C.et al.Correlation between NT-proBNP and 25(OH)VD, cTnT and hs-CRP in patients with chronic heart failure complicated with chronic kidney disease. *Chinese Medical Science*. **8**, 71-74 (2018).
60. Hui,C.et al. To observe the level of serum 25-hydroxyvitamin D in elderly patients with osteoporosis complicated with type 2 diabetes. *Imaging Research and Medical Application*. **2**, 254-255 (2018).
61. Ling G.et al. Value of serum 25-hydroxyvitamin D and tumor markers in the diagnosis of breast malignant tumor. *Geriatrics and Health Care*. **24**, 587-588 (2018).
62. Yunyun, J.et al. Correlation analysis between hypothyroidism and bone mineral density in middle-aged and elderly women. *labeled immunoassays and clinical medicine.* **25**, 454-456 (2018).
63. Shize,L. et al. Correlation between serum 25- hydroxyvitamin D3 level and the severity of community-acquired pneumonia complicated with sepsis. *Journal of Taishan Medical College.* **39**, 789-791 (2018).
64. Zhiyuan. L. Analysis of serum 25- hydroxyvitamin D level and its influencing factors in patients with severe pneumonia. *Journal of Internal Medicine Critical Care*. **24**, 428-430 (2018).
65. Guangxiao, N. et al. Correlation between serum 25- hydroxyvitamin D level and ankle bone marrow edema syndrome. *Journal of Hebei Medical University*. **39**, 1334-1338 (2018).
66. Dagang D.et al. Re-study on insulin-like growth factor 1 and 25-hydroxyvitamin D and female essential hypertension. *Chinese Medical Innovation*. **15**, 15-19 (2018).
67. Zongzhe, X.et al. Study on the correlation between serum 25- hydroxyvitamin D level and lung cancer. *Journal of Clinical Military Medicine*. **46,** 702-704 (2018).
68. Qigang, Y.et al. Effect of vitamin D_2 on serum 25- hydroxyvitamin D in elderly women. *Chinese Journal of Clinical Pharmacology*. **34**, 640-642 (2018).
69. Xia, Y.et al. Correlation analysis of calmodulin, bone turnover markers and bone mineral density in postmenopausal patients with type 2 diabetes. *Ningxia Medical Journal*. **40,** 772-775 (2018).
70. Yoverdin. Effect of long-term vitamin D supplementation on coronary artery calcification in patients with type 2 diabetes. *Chongqing Medical*. **47**, 3529-3532 (2018).
71. Lei Z.et al. Correlation analysis of nail fold microcirculation score with disease activity and osteoporosis in patients with early rheumatoid arthritis. *Chinese Journal of Osteoporosis*. **24**, 1021-1028 (2018).
72. Xin, Z.et al.Analysis of serum vitamin D level in middle-aged and elderly patients with diabetes, hypertension and dyslipidemia. *Chinese Journal of Geriatrics*. **37**, 764-767 (2018).
73. Yan, Z.et al. Correlation between serum vitamin D level and quality of life and inflammatory factors in elderly patients with chronic obstructive pulmonary disease. *Geriatrics and Health Care*. 24, 408-411 (2018).
74. Fan, Z. Clinical study of Shensong Yangxin Capsule combined with vitamin D in adjuvant treatment of essential hypertension. *New Traditional Chinese Medicine*. **50,** 23-25 (2018).
75. Rui Xiang, C.et al. Analysis of serum 25- hydroxyvitamin D level and related factors in middle-aged and elderly patients with type 2 diabetes. *Journal of Tongji University (Medical Edition)* .**38,**84-88 (2017).
76. Xi, C.et al.Investigation and analysis of mineral and bone metabolism indexes in patients with diabetic nephropathy. *Chinese Journal of Integrated Traditional and Western Nephrology.* **18,** 1074-1076 (2017).
77. Wei, C.et al. Study on the relationship between serum 25- hydroxyvitamin D, vitamin D binding protein, Treg cells and clinicopathological features in patients with ovarian cancer. *Medical Theory and Practice*. **30**, 487-488 (2017).
78. Jixing, F.et al. Correlation analysis of serum 25(OH)D and bone turnover markers with hip fracture in the elderly. *Chinese Journal of Osteoporosis*. **23,** 1451-1456 (2017).
79. Houying, F.et al. Analysis of serum 25- hydroxyvitamin D level and the severity of acute ischemic stroke. *Chinese Journal of Practical Nervous Diseases*. **20,** 40-43 (2017).
80. Pan, H.et al.Correlation between Alzheimer's disease and serum vitamin D and blood calcium levels. *Medical Theory and Practice*. **30,** 2670-2672 (2017).
81. Rongxiang. L. Correlation between serum 25- hydroxyvitamin D level and coronary artery stenosis in patients with coronary heart disease in Qinghai [Master]: *Qinghai University*, 2017.
82. Xiangfei,P.et al. Correlation between serum vitamin D level, Cat score and inflammatory factors in patients with acute exacerbation of chronic obstructive pulmonary disease. *Chinese and Foreign Medical Research*. **15,** 74-75 (2017).
83. Mei ,Q. Study on the application of knee joint ultrasound and serum 25- hydroxyvitamin D level in patients with knee osteoarthritis [Master]: *Qinghai University*, 2017.
84. Shenlin, W.et al. Study on the relationship between vitamin D and bacterial colonization of lower respiratory tract and phlegm-related inflammatory factors in Copd patients. *Ningxia Medical Journal*. **39,** 964-967 (2017).
85. Huimin,X.et al. Study on the correlation between serum 25-hydroxyvitamin D, homocysteine and apolipoprotein B/A1 levels and patients with essential hypertension. *Chinese Journal of Health Laboratory Technology*. **27**, 1448-1450 (2017).
86. Taoping, Y.et al. Study on the correlation between vitamin D level and blood routine in Copd patients. *Chinese Community Physician*. **33**, 130-131 (2017).
87. Kezhao, Z. Clinical application of 25-hydroxyvitamin D and ferritin in type 2 diabetes. *Experimental and Laboratory Medicine.* **35**, 734-736 (2017).
88. Qinfang, Z.et al. Effect of calcium and vitamin D supplementation on bone mineral level and body composition of elderly postmenopausal women. *Laboratory Medicine and Clinical*. **14**, 3305-3308 (2017).
89. Gong Y.H, Chen Suzhen & Ding Haiming. Changes and significance of serum 25- hydroxyvitamin D concentration in patients with type 2 diabetes. *Modern Medicine and Health*. **32**, 1622-1623 (2016).
90. Yinshan. H. Correlation between bone mineral density and 25- hydroxyvitamin D3 in patients with rheumatoid arthritis [Master]: *North Sichuan Medical College,* 2016.
91. Shaohui, S.et al.Study on the correlation between serum 25-hydroxyvitamin D and knee osteoarthritis pain. *Chinese Contemporary Medicine*. **23**, 67-70 (2016).
92. Changhong, S. Study on the correlation between first-onset type 2 diabetes and serum 25- hydroxyvitamin D level. *Diabetes New World*. **19**, 53-54 (2016).
93. Siming,W.et al. Relationship between serum 25- hydroxyvitamin D level and subclinical atherosclerosis in the elderly. *Chinese Journal of Geriatrics*. **35**, 1201-1205 (2016).
94. Jiajia,W.et al. Related Factors of Myopenia in Patients with Type 2 Diabetes Mellitus. *Chinese Journal of Osteoporosis and Bone Mineral Diseases.* **9**, 129-135 (2016).
95. Qian, X.et al. Relationship between serum vitamin D and parathyroid hormone levels and the product of heart rate and systolic blood pressure. *Journal of Nanjing Medical University (Natural Science Edition)* .**36**,234-238 (2016).
96. Yanpeng, Y. Clinical study of vitamin D in peripheral blood of patients with bronchiectasis [Master]: *Shandong University*, 2016.
97. Quanli,Z. Correlation between serum vitamin D level and quality of life of Copd patients and inflammatory factors related to Copd. *Journal of Clinical and Experimental Medicine*. **15**, 665-668 (2016).
98. Xiaojing, C.et al.Correlation between vitamin D receptor gene polymorphism and chronic periodontitis. *Journal of Peking University (Medical Edition)*. **47**, 697-702 (2015).
99. Yijing ,L. Study on the correlation between 25- hydroxyvitamin D and rheumatoid arthritis [Master]: *Jilin University*, 2015.
100. Jinfang, Q.et al. Effect of serum 25- hydroxyvitamin D3 in the third trimester of pregnancy on insulin resistance and bone metabolism. *China Modern Doctor*. **53**, 16-18 (2015).
101. Qihong ,Q. Discussion on the value of bone metabolism index in the diagnosis of primary osteoporosis [Master]: *Zhejiang University*, 2015.
102. Qihong, Q. Wan Shuanglin & Ma Yan. The value of bone metabolism index in the diagnosis of primary osteoporosis. *Clinical Education of General Practice*. **13**, 144-146 (2015).
103. Xiaokai, S.et al.Clinical study on the relationship between 25- hydroxyvitamin D and cerebral infarction and intervention treatment. *Chinese Journal of Stroke*. **10**, 231-237 (2015).
104. Wang,H. Study on the correlation between serum 25- hydroxyvitamin D level and blood pressure variability in patients with essential hypertension [Master]: *Xinjiang Medical University*, 2015.
105. Meiqin, W.et al. Study on the correlation between the polymorphism of vitamin D binding protein gene Rs2282679 A/C and chronic obstructive pulmonary disease. *Chinese General Medicine*. 18, 3678-3681 (2015).
106. Ting, W.et al.Analysis of changes of Pth and 25-Oh-Vd in patients with premature ovarian failure and osteoporosis. *Chinese Journal of Woman and Child Health Research*. **26**, 694-696 (2015).
107. John, Y. Analysis of Gender Differences and Related Factors of Bone Mineral Density Changes in Epilepsy Patients [Master]: *Tianjin Medical University*, 2015.
108. Wenbin, Y.et al. Correlation between subcortical arteriosclerosis encephalopathy and vitamin D level. *Modern Hospital*. **15**, 36-37 (2015).
109. Yanxia, Y.et al. Correlation between bone mineral density and bone metabolism indexes in patients with osteoporosis. *China Modern Doctor*. **53**, 80-82 (2015).
110. Fang, Z.et al. Study on changes of serum bone metabolism indexes of osteoarthritis. *Journal of Xinjiang Medical University*. **38**, 1492-1495 (2015).
111. Xiaokai,S. Clinical study on the relationship between 25- hydroxyvitamin D and ischemic stroke and intervention therapy. *7th National Academic Conference on Neurology for Young and Middle-aged People of Neurology Branch of Chinese Medical Association and 10th National Academic Conference on Infectious Diseases of Nervous System and Cytology of Cerebrospinal Fluid. Xi 'an, Shaanxi, China*, 2014:171.
112. Xiaokai, S.et al. Clinical study on the relationship between 25- hydroxyvitamin D and ischemic stroke and intervention treatment. China Cerebrovascular Disease Congress 2014. *Changsha, Hunan, China*, 2014:320.
113. Maofeng, W.et al. Clinical significance of plasma interleukin-10 and 25- hydroxyvitamin D in patients with lung cancer. *Journal of Wenzhou Medical University*. **44**, 829-831 (2014).
114. Ying,W.et al. Analysis of bone metabolism balance in chronic renal failure patients with 25- hydroxyvitamin D deficiency. *The First National Conference on Laboratory Medicine of Integrated Traditional Chinese and Western Medicine and the Inaugural Meeting of Laboratory Medicine Committee of Chinese Society of Integrated Traditional Chinese and Western Medicine. Beijing, China*, 2014:321-324.
115. Yanli, X.et al.Analysis of clinicopathological features of colorectal cancer and its correlation with serum 25-hydroxyvitamin D. *Gastroenterology*. **19,** 156-160 (2014).
116. Xiaojuan, F.et al. Changes and significance of serum 25-hydroxyvitamin D and Crp in patients with diabetic nephropathy. *Journal of Clinical Laboratory*. **31**, 838-840 (2013).
117. Qiong ,H.et al. Analysis of risk factors of osteoporosis in patients with rheumatoid arthritis. *Journal of Anhui Medical University*.**48**,1083-1087 (2013).
118. Qiong, H.et al. Changes of serum 25-hydroxyvitamin D level in patients with rheumatoid arthritis and its clinical significance. *Chinese Journal of Rheumatology*. **17**, 159-163 (2013).
119. Encai,L. Study on the correlation between bone metabolic diseases and 25- hydroxyvitamin D3 in the elderly in Hulunbeier area. *journal of hulunbeier college*. **21**, 112-113 (2013).
120. Yelan, R. Correlation between vitamin D and fibrinogen in essential hypertension [Master]: *Xinjiang Medical University*, 2013.
121. Wenhua,P.et al. Correlation between serum 25-hydroxyvitamin D level and acute coronary syndrome. *Chinese Journal of Osteoporosis and Bone Mineral Salt Diseases*. **5**, 100-104 (2012).
122. Xiuzhen, Z.et al. Study on biochemical indexes of bone turnover in postmenopausal women with type 2 diabetes. *Chinese Journal of Osteoporosis and Bone Mineral Diseases*. **5,** 30-34 (2012).
123. Yongfang,Z. Analysis of 25- hydroxyvitamin D and blood lipid levels in elderly patients with type 2 diabetes. *practical geriatrics*. **26**, 323-325 (2012).
